# Pharmacists’ perceived role in supporting diabetes education and self-management in Ireland: a qualitative study

**DOI:** 10.12688/hrbopenres.13192.1

**Published:** 2021-02-18

**Authors:** Eva Cooney, David O'Riordan, Jennifer McSharry

**Affiliations:** 1 Health Behaviour Change Research Group, School of Psychology, National University of Ireland, Galway, Galway, Ireland; 2 School of Public Health, University College Cork, Cork, Ireland

**Keywords:** Diabetes Mellitus, Pharmacist, Self-management, Qualitative Research, Education, Health Care Delivery.

## Abstract

**Background: **Support for people with diabetes is necessary for optimal self-management. Structured diabetes education programmes fulfil this need, but attendance rates are consistently low. The role of pharmacists has expanded but the profession remains underutilised in chronic disease management. The objective of this study is to explore pharmacists’ perceived role in the support of diabetes education and self-management behaviours.

**Methods:** A qualitative study using semi-structured interviews of community pharmacists in Ireland was conducted. Interviews were audio-recorded, transcribed verbatim and analysed using inductive thematic analysis.

**Results:** Ten pharmacists were interviewed. The four themes identified illustrate the juxtaposition of pharmacists’ potential in diabetes care with the realities of current pharmaceutical practice. One theme outlined the relationship between the person with diabetes and the pharmacist, ‘Patient or customer: the nature of the pharmacist relationship’. Two themes described the pharmacists’ role in supporting diabetes education and self-management, ‘Beyond medication: pharmacists’ current and potential role in diabetes management’ and ‘Need for diabetes education’. The final theme highlighted the barriers to a more engaged role in patient care, ‘Barriers: “all the stuff that gets in the way”’.

**Conclusion:** The relationship between pharmacists and people with diabetes could facilitate pharmacists in supporting diabetes self-management. However, variability across pharmacists’ level of involvement and consistent resource barriers were noted. Pharmacists were poorly informed about structured diabetes education programmes. Further research is needed to explore this variability but there may be potential to enhance the pharmacist role in promoting attendance at structured diabetes education programmes.

## Introduction

Optimal management of diabetes improves quality of life and minimises the cost of diabetes for the individual and the healthcare system. Clinical care for people with diabetes has been described as minimal and disjointed, with people with diabetes sometimes seen just once a year ^1^. As such, it is crucial that people with diabetes are equipped with the skills, knowledge and support to self-manage their condition. However, adherence to self-management plans is often suboptimal, resulting in poorly managed diabetes thus increasing the risk of macrovascular and microvascular complications ^2^. In response to this, structured diabetes education (SDE) programmes have been developed and implemented internationally ^3^, with increasing focus on empowering people with diabetes to self-manage their health ^4^.

Attendance at SDE has been linked to improvements across clinical, lifestyle and psychosocial outcomes ^5^ and is cost-effective relative to usual care ^6^. International guidelines have recommended SDE as a core component of the diabetes care pathway ^7^.

Despite this, poor programme uptake is a significant challenge and has been identified as a research priority by people with diabetes, practitioners and policy makers ^8^. While reasons for non-attendance are diverse and complex ^9^, healthcare professional (HCP) communication about SDE has been identified as influential ^10^. Research has suggested that exploring and improving HCP knowledge of SDE benefits may facilitate improved awareness and subsequent attendance at SDE by people with diabetes ^10^.

### The role of pharmacists

One HCP group with a potential role in supporting SDE attendance and diabetes self-management are pharmacists. An evolution of the pharmacist role from a singular focus on medication dispensing to more patient-centred, collaborative care is evident in the literature ^11– 13^. Concerns about the sustainability of healthcare expenditure and increasing demands on primary care physicians have fuelled this movement ^14^. Several countries have expanded the role of pharmacists to facilitate coordinated care delivery, such as provision of emergency prescription refills and administration of vaccinations ^14^, and in the guidance, education, and support of people with chronic disease ^15^. Community pharmacists play a vital role in the primary healthcare system in Ireland. Some of the health services provided by pharmacists include administration of the flu vaccine, blood pressure measurement, cholesterol testing, smoking cessation, and provision of emergency contraception. However, services such as medicine use reviews, minor ailment schemes, and chronic disease management services are currently not provided in a pharmacy setting. A recent review found that pharmacists see people between 1.5 and 10 times more frequently than general practitioners (GPs) ^16^. In Ireland, it is estimated that 2 million people visit a community pharmacy each month ^17^. People with diabetes have regular contact with their pharmacist, identifying it as a condition in which pharmacists could assume a greater role in care ^18, 19^.

Evidence from meta-analyses has highlighted the effectiveness of diabetes self-management support delivered by pharmacists in improving adherence, glycaemic control and diabetes knowledge when compared to usual care ^20, 21^. There is also said to be considerable cost savings from greater utilisation of pharmacists in diabetes support ^22^. However, the same authors highlight the issue of inadequate reimbursement mechanisms for clinical pharmacy services. Lack of resources has also been identified as a challenge to pharmacists assuming a more active role in diabetes care ^11, 12^.

In Ireland, national frameworks highlight the need to provide self-management support for chronic illness within routine care ^23^and Irish research on Type 2 diabetes SDE attendance identified the need to look beyond the attitudes of people with diabetes, to explore the role of HCPs in supporting SDE attendance ^10^. Existing qualitative research has explored the role of pharmacists in diabetes self-management support from the perspective of people with diabetes ^12, 18^ but there is a paucity of research regarding pharmacists’ own views on the topic.

### Aim of the study

The current study aims to develop a greater understanding of the potential role of pharmacists in supporting people with Type 1 and Type 2 diabetes. Specifically, this study will explore pharmacists’ knowledge of SDE and their perceived role in diabetes education and self-management in Ireland.

## Methods

The COnsolidated criteria for REporting Qualitative research (COREQ) checklist ( *Extended data* ^24^) was used to guide conduct and reporting of a semi-structured qualitative interview study ^25^. Semi-structured qualitative interviews were chosen as they have been found to provide deeper insight to an individual’s experiences, perceptions and opinions compared to other qualitative methods ^26^.

### Ethics approval

Ethical approval was granted by the National University of Ireland Galway (NUIG) School of Psychology Research Ethics Committee in January 2019 (SREC 24/01/2019).

### Participants and recruitment

Community pharmacists currently working in Ireland were eligible for this study. Invitations to participate were circulated through the Irish Pharmacy Union (IPU, the representative and professional body for community pharmacists in Ireland), PharmaBuddy (an online pharmacist support group), on Facebook and delivered to pharmacies in the West and Midlands of Ireland. All invitations included an overview of the study and an email address to express interest in participation and to receive an information sheet and consent form. Convenience sampling was utilised, and all interested participants were interviewed. Within the context of the current study, the data collected was judged to reach saturation by the research team defined as data being adequate in both amount and variety to answer the research question ^27^.

### Data collection

Semi-structured interviews were conducted by EC, a female student health psychologist (B.A Psychology), between February and June 2019. EC worked with an experienced qualitative supervisor (JMS) in completing the study. EC communicated with participants prior to the interview to inform them about the study, receive written informed consent for their participation and to arrange an interview time. There was no previous relationship between the interviewer and participants, participants were informed that the study was being carried out for a MSc dissertation, but no other researcher characteristics were provided.

Interviews were held face-to-face and over the phone, with only the interviewer and participant present. EC conducted phone interviews (n = 8) in a private room while interviews held in person (n = 2) were conducted in an office and pharmacy consultation room. A topic guide of open-ended questions (see *Extended data* ^24^) focused on the pharmacist role in supporting Type 1 and Type 2 diabetes self-management behaviours and SDE attendance was used flexibly to guide each interview. No field notes were taken as interviews were primarily held by phone and were audio recorded. Prompts were included to probe answers where necessary. This guide was developed based on previous literature on pharmacists’ roles in patient care ^13^, diabetes self-management and SDE attendance ^10^.

All interviews were audio-recorded, transcribed verbatim and checked for accuracy by EC. Following the first two interviews, the transcripts were reviewed by EC and JMS. The validation technique of member checking was offered to all participants; however, this was declined. Transcripts were anonymised and imported to the software package NVivo 12 to facilitate data organisation, management and analysis.

### Data analysis

Interviews were coded alongside data collection. Data were analysed following an inductive reflexive thematic analysis approach ^28^ and according to a critical realist framework as posited by Maxwell ^29^.

Line by line coding was carried out using NVivo by EC. Initial conceptual themes and codes were identified and applied to subsequent transcripts and were modified and developed as necessary. Data saturation was reached at analysis of the final two interviews. Draft themes were reviewed by JMS alongside transcripts and discussed with EC. A selection of extracts were chosen to illustrate each theme. Extracts were edited for clarity ^30^ and identified by a participant number. Participants were not involved in the analysis process. A summary of findings was sent to all participants following analysis.

## Results

In total, 15 pharmacists expressed interest in this study; 10 were available for interview during the data collection period (IPU: 1, PharmaBuddy: 3, Facebook: 6). Interviews lasted between 11 and 45 minutes ( *M* = 21 minutes). Eight interviews took place over the phone and two face-to-face. Participant demographic characteristics were collected at the beginning of each interview and can be found in Table 1.

**Table 1.** Participant demographics (n=10).

	*n*
Gender	
Male	4
Female	6
Years of practice as a pharmacist	
<5	2
6–10	1
11–15	4
16–20	0
21+	3
Pharmacy type	
Independent	7
Group	1
Chain	2
Pharmacy location	
Rural	2
Suburban	3
Urban	5

Note: *n* = number of participants.

Four themes were identified, two of which have two sub-themes, as shown in Table 2. The first theme provided a foundation from which to understand the pharmacist role in diabetes care, ‘Patient or customer: the nature of the pharmacist-patient relationship’. The second theme described the pharmacist role in wider diabetes management, ‘Beyond medication: pharmacists’ current and potential role in diabetes management’ and included two sub-themes, which outlined the disparity between pharmacists’ current and potential roles. The third theme, ‘Need for diabetes education’, included two sub-themes that highlighted the pharmacists’ belief in the importance of knowledge for diabetes management and the ambiguity around sources of education and information. The final theme, ‘Barriers: “all the stuff that gets in the way”’ outlined the issues that prevent pharmacists from greater involvement in education and support of diabetes self-management.

**Table 2.** Themes and subthemes identified.

Themes	Sub-themes
Patient or customer: the nature of the pharmacist relationship	
Beyond medication: pharmacists current and potential role in diabetes management	Pharmacists’ current role Pharmacists not “utilised as best they should be”
Need for diabetes education	The importance of patient knowledge The availability of patient education
Barriers: “all the stuff that gets in the way”	

### Patient or customer: the nature of the pharmacist relationship

The nature of the pharmacists’ relationship with, and the terms used to describe, the people who use their services depends on the transaction type, but pharmacists primarily used the term patients. As such, this term is used to refer to people with diabetes within the results section.

*“Now if someone is just coming in to buy tan or something, they’d be customers but if we have any role in their care at all we would say patients.” (Participant 2)*

Pharmacists’ highlighted their accessibility compared to other HCPs as they are available in the evenings and at the weekend. With no need to make an appointment they tend to be the *“first port of call” (Participant 3, 6 & 8)*. Pharmacists believed that they had more contact with people with diabetes than other HCPs and described themselves as being *“the only person in the whole system that knows the full picture” (Participant 8).* This frequency of contact facilitates a level of familiarity and honesty within the relationship.

*“A pharmacist has more touch points with a diabetic patient than any other member of the healthcare system and it'd be very common that the patient would be more honest with the pharmacist than their doctor or their endocrinologist or diabetic nurse.” (Participant 4)*

Pharmacists positioned themselves as having a close and caring relationship with their patients, emphasising the importance of a personal connection. However, one pharmacist believed it was not the familiarity but the profession which garners this respect.

*“There can be a build-up of confidence in knowing somebody from repeated visits. But I think pharmacists are regarded with some degree of trust.” (Participant 9)*

Pharmacists believed that patients’ perception of the pharmacist role within the wider healthcare team also influences their relationship. Pharmacists explained that some patients believe that the pharmacist role is solely medication dispensing and consequently will not engage beyond this.

*“It depends a lot on how the patient sees you. Some of the time you’re quite involved in their care and you would talk through a lot of things. Others, they don't want to hear from you, they just want to talk to a doctor.” (Participant 10)*

### Beyond medication: pharmacists current and potential roles in diabetes management

Pharmacists emphasised their role in medication dispensing as the *“gatekeepers of medicines” (Participant 8)*, but also acknowledged a wider and *“strong community based healthcare role” (Participant 4)*. There were two aspects in pharmacists’ discussion of their role in wider diabetes care: what they currently do and what they could do. As such, two sub-themes were identified: pharmacists’ current role and pharmacists “not being utilised as best they should be”.

***Pharmacists’ current role.*** The level of involvement in wider diabetes care varied across pharmacists interviewed. There was a belief that people with diabetes are *“not just there to be given the medication ... but also to be educated on their disease so that they themselves are able to manage their disease better” (Participant 1).* The presence of the pharmacist as a source of motivational support for patients was also described:

*“No matter what medication you put people on, if they are not looking after and motivated to be involved in it themselves, they will have poor outcomes.” (Participant 4)*

While pharmacists would engage in advice on diabetes self-management, some would not do so unprompted.

*“If people ask me about it I would help them, but in terms of having a proactive approach to it, I never have really.” (Participant 2)*

It appeared that there is no standardisation or structure in place for engagement with patients on the wider management of their conditions.

*“And in terms of structured interventions, that wouldn't really be the format that they take. Sometimes they say can I have a chat with you and you make the time, go into the consultation room and have a discussion for five minutes or so.” (Participant 8)*

This lack of structure may underlie the differences in opinion across pharmacists regarding the support of diabetes self-management. One pharmacist perceived no role in supporting diabetes’ self-management, *“Generally, I think most of that goes through the diabetic clinics and the doctor” (Participant 9),* describing their role as sourcing and supplying medication rather than informing.

***Pharmacists not “utilised as best they should be”.*** Pharmacists felt that they are limited in what they can currently do and are not *“being utilised as best as they should be.” (Participant 3).* The primary area in which pharmacists described the potential to assume a greater role was in advising on and adjusting medication. Referring to the precedent set by their counterparts in other jurisdictions, pharmacists proposed that they could enhance patient care and reduce the need to visit a GP, freeing up services in certain scenarios:

*“And we do know more about the drugs and how to interchange them and how a little bit of tweaking can make a big difference, without a person having to go to the GP or wait and see the consultant or clog up the services.” (Participant 7)*

One pharmacist also believed there was scope for developing specific lifestyle support, *“like diet plans or weight management that would all fall in under the broad umbrella of diabetes” (Participant 5).* However, pharmacists noted that patients may not take advantage of the expertise of pharmacists beyond medication: “ *When you start to talk about lifestyle and exercise you sort of get a ‘yeah, yeah grand’” (Participant 10).*

### Need for diabetes education

Pharmacists were clear in their belief that education is a key component of diabetes management. Two sub-themes were identified: the importance of patient knowledge and the availability of patient education.

***The importance of patient knowledge.*** Pharmacists described that patients differed in their knowledge about diabetes. Several issues are apparent when knowledge is insufficient, with one pharmacist describing patients who fail to *“realise that they’re diabetic or would think that things [anti-inflammatory medication] had caused diabetes” (Participant 3).* A lack of patient knowledge on how lifestyle interacts with diabetes was also noted by pharmacists: *“people are not aware of how their lifestyle ... especially where diet is concerned, it can contribute to the worsening of the condition” (Participant 1).* Pharmacists believed that some people with diabetes do not realise the severity of their condition and thus are slow to adjust their lifestyle, relying instead on increasing medication.

*“I don’t think people realise they can lose fingers, they can lose toes and their eyesight can go ... they’re not scared by it, because everybody has it. It’s just an extra tablet, they don’t realise the seriousness of it. They really don’t.” (Participant 7)*

Pharmacists suggested that diabetes medication non-adherence and misunderstanding regarding its function, or a perceived lack of benefits, may also result from a lack of knowledge.

*“...if they don't understand what they're for, often they don't appreciate the importance, they don’t take them.” (Participant 8)*

However, the potential to educate patients and the benefits of this were evident. Pharmacists acknowledged the growing emphasis on education for management: “ *Definitely the education around the carbohydrate counting and stuff is where it’s going in terms of management” (Participant 6)*, and in empowering and motivating patients: *“once they feel more educated, they feel more involved, which is definitely beneficial” (Participant 10).*

***Availability of patient education.*** Closely linked to the importance of diabetes knowledge was the availability of education for people with diabetes. Pharmacists discussed various sources of patient education. One pharmacist suggested that patients often seek advice on patient-driven forums: *“a lot of it is ill informed, patient driven, more so than healthcare driven” (Participant 4)*. Another described educating patients about aspects of their care because the wait for a diabetic clinic appointment is *“...just ridiculous. By the time they get an appointment with the diabetic clinic they could be up to three tablets” (Participant 3).* Further, one pharmacist holds information days to supplement their patients’ diabetes care:

*“I’ll get somebody from the heart foundation or the diabetes foundation … they’re great days actually and people love coming to them to get seen and someone can chat them and give them a bit of time.” (Participant 7)*

Pharmacists referenced several established services for diabetes, but SDE was only mentioned unprompted by one pharmacist and most were unaware of the existence of SDE programmes.

*“I have never heard of those and I have never come across any patient that has ever attended such a course.” (Participant 3)*

Those who had heard of SDE had limited knowledge *: “well I know that they exist somewhere but if someone asked me to refer them, I’d have to google it” (Participant 2).* However, there was interest in the programmes and a view that pharmacists could promote SDE to their patients, “ *because it’s just such an ease of access, it makes sense to have something there that you could just keep reminding them” (Participant 10),* with suggestions such as inclusion of a leaflet with their prescriptions *“to bring awareness that there are education classes available” (Participant 1).*

### Barriers “all the stuff that gets in the way”

Despite pharmacists’ interest in engaging in discussions around diabetes self-management, several barriers were described. The predominant barrier was resources; time, pharmacists and finance, all of which were closely linked. Time constraints were mentioned by several pharmacists due to an increase in paperwork and administration.

*“So you don't have a lot of free time to go out and talk to people and spend as much time with them as you’d like to.” (Participant 6)*

Time to engage in patient consultation was also compromised by *“pressure to supervise other services in the pharmacy” (Participant 1),* highlighting the issue of staffing. However, the cost of having a second pharmacist often cannot be sustained because the *“numbers of prescriptions doesn’t constitute enough for a second pharmacist” (Participant 5)*. This reflects the structure of pharmacy services and what the pharmacists are paid for.

*“The big thing that stops pharmacists having a far more engaged role is the way it's set up in terms of what pharmacists are actually paid for.” (Participant 8)*

## Discussion

This study explored Irish pharmacists’ perceived role in the support of people with diabetes self-management and education. Four themes were identified which illustrate the juxtaposition of pharmacists’ perceived roles in diabetes care with the realities of current pharmaceutical practice: ‘Patient or customer: the nature of the patient relationship’, ‘Beyond medication: the current and potential roles of pharmacists’, ‘Need for education’ and ‘Barriers: “all the stuff that gets in the way”’. The first three themes fulfil the aims of this study. However, the fourth theme highlights that pharmacists’ interest in assuming a greater role in diabetes care may not be realistic given the current practice structure.

It was apparent that pharmacists play an integral role in the care of people with diabetes. The relationship between pharmacists and people with diabetes and the honesty cultivated through frequency of contact and familiarity was key to this role. Contrary to Twigg *et al.* ^18^ finding that people did not view pharmacists as their first access point for advice, the pharmacists in this study portrayed themselves as an accessible, first point of contact. The discussion of the evolution of the pharmacist role through the expansion of services and broader care was reflective of the wider literature ^11– 13, 18^. However, the current study identified inconsistencies in this expanded role across pharmacists and whether it was prompted by the pharmacist or the person with diabetes. There appeared to be a lack of clear guidance leaving individual practices to decide what extended services are offered. Furthermore, there was a disparity in the pharmacists’ current and potential roles in diabetes care. Several pharmacists suggested that they are underutilised, particularly when comparing themselves to counterparts in other jurisdictions. Pharmacists suggested that if given the opportunity to assume more responsibility in adjusting types and doses of medication, they could enhance the care received by people with diabetes and reduce the burden on other healthcare services.

Most pharmacists were unfamiliar with SDE. It remains unclear why this awareness was lacking but this aligns with the findings of previous research which highlighted the lack of HCP knowledge and communication around SDE ^10^. Lack of awareness of SDE has important repercussions for the attendance of people with diabetes at SDE, as pharmacists cannot encourage attendance at programmes they are not aware of.

The fourth theme, ‘Barriers: “all the stuff that gets in the way’, emphasised the reality of pharmaceutical practice. Despite pharmacists’ interest in more engaged care, there are several barriers. This largely pertains to the difficulty of balancing consultation with supervising prescription sales. The barriers identified in this study were consistent with those previously identified in the literature; time, lack of resources and a high workload ^11, 12^. This study would support the proposition that the role of pharmacists could be enhanced and encouraged with a broader scope of practice and appropriate remuneration ^14, 16^. This highlights the need for policy makers to engage with pharmacists in discussion around the expansion of pharmacy services. The potential for success in such expansions are apparent in the results of pharmacists delivering the flu vaccination with higher immunisation rates and general cost savings ^31^.

A strength of this study is the insight provided into pharmacists’ own perception of their role in supporting diabetes self-management behaviours, a topic under-explored in existing literature. Due to the variation in pharmacists’ roles across jurisdictions, an exploration in the Irish context is also important. The use of qualitative methodology provided greater depth than could have been achieved through a quantitative study. The open-ended questions allowed the pharmacists to talk about the topics freely and facilitated adjustment of the topic guide where necessary.

Limitations to this study are acknowledged. Firstly, although it was intended that maximum variation sampling be used, this was not possible. In hindsight, purposive sampling may have been more appropriate to pursue for this study. Despite the reliance on convenience sampling, participants did vary across key demographics, as can be noted in Table 1. All pharmacists interviewed were Irish and so the findings cannot be generalised beyond the Irish context. The Irish perspective may be of benefit to the international discussion on the role of pharmacists in community practice and this topic would benefit from additional research exploring and comparing international perspectives. Overall, the number of interested participants was low which may indicate a lack of interest in the topic, or general reluctance toward research participation given the time pressure on pharmacists. The pharmacists who chose to take part may have had a particular interest in diabetes and self-management support which may have impacted the findings.

Further research is required to investigate the variability of involvement in diabetes self-management support across pharmacists and to enhance and standardise the pharmacist role. The findings indicate that pharmacists had limited to no knowledge of SDE but believed such programmes would be beneficial. Given the findings of previous research on the barriers to SDE attendance ^10^, this exemplifies the potential for pharmacists to assume a greater role in promoting SDE. This would require minimal resources, as pharmacists themselves identified the potential to include an SDE leaflet with monthly prescriptions. However, more research is first required to identify why pharmacists are unaware of SDE and how this can be addressed.

There is international discussion of the expanding role of the pharmacist, however, this role varies widely across countries due to legislative differences in pharmacy practice. For example, pharmacists in the United Kingdom can specialise in clinical areas such as diabetes and work in general practice as independent prescribers ^32^. The broader findings of this study regarding the barriers of resources and reimbursement structures, and the potential role for pharmacists in medication adjustment also require further research, but indicate that significant, structural and legislative changes may be needed. Progress is being made in Ireland and research has highlighted the potential for pharmacists working within general practice in Ireland to improve prescribing quality and optimise clinical care ^33^.

## Conclusion

It would be naïve to recommend a greater role for pharmacists in chronic disease management without highlighting the current barriers faced by the profession. This study investigated Irish pharmacists’ perceived role in the support of people with diabetes, highlighted the variation and potential in pharmacy practice and identified barriers to more engaged care. This adds to the international literature on pharmacist interventions for diabetes self-management but also creates a basis from which the role of Irish pharmacists can be further researched and advanced. The prevention and minimisation of diabetes related complications is paramount for global health, and the current research is a starting point from which the role of pharmacists in enhancing diabetes care can be further investigated.

## Data availability

### Underlying data

There are no quantitative data associated with this article. The audio files and transcripts generated during the current study are confidential. In the consent document signed by participants it stated that ‘no-one but the research team would hear the recordings’.

As a result, researchers seeking to access the underlying data (i.e. audio files and transcripts) will need to apply directly to the NUI Galway Research Ethics Committee for approval. The Committee can be contacted at ethics@nuigalway.ie. Should approval be granted, the authors are happy to facilitate access. Quotes reflecting the transcripts are available in the article itself.

### Extended data

Open Science Framework: Pharmacists’ perceived role in supporting diabetes education and self-management in Ireland: a qualitative study, https://doi.org/10.17605/OSF.IO/B289U ^24^. 

This project contains the following extended data:

- Topic guide
- COREQ checklist

Data are available under the terms of the Creative Commons Zero "No rights reserved" data waiver (CC0 1.0 Public domain dedication).
