# Supplementary material for: Pharmacists’ perceived role in supporting diabetes education and self-management in Ireland: a qualitative study
Source: HRB Open Res. 2022 Apr 11;4:20. Originally published 2021 Feb 18. [Version 2] doi: 10.12688/hrbopenres.13192.2 (PMC8546734; doi:10.12688/hrbopenres.13192.2)
Supplement: Peer review history

# Peer review: Reviewer response for version 1

**DOI:** 10.21956/hrbopenres.14335.r30554

**Published:** 2021-10-25

**Authors:** Hanni Puspitasari

**Affiliations:** 1 Department of Pharmacy Practice, Faculty of Pharmacy, Universitas Airlangga, Surabaya, Indonesia

This is a great and interesting article to describe pharmacists’ perspective in the care of patients with diabetes. There are, however, some notes I made.

1. In the “Participants and recruitment” section: It is not clear how the judgment was made to decide that data saturation was reached. It seems that invitations were circulated at once, then interviews were conducted after obtaining all participants. If so, how did the researchers define "saturation" from 10 interviews? It would be nice if the authors describe the recruitment and data collection process to finally reach data saturation.
2. In the “Results” section: To give better understanding about participating pharmacists, it would be better if the total number of invitations is stated, so the audience can get the impression of the response rate.
3. In the “Patient or customer: the nature of the pharmacist relationship” section, second paragraph: Is the subject of this sentence "pharmacists"? If so, it shouldn’t use an apostrophe(‘).
4. In the “Availability of patient education” section, on the first quote: It is not clear what "they" in "they're great days" refers to.
5. In the “Discussion” section, 7 ^th^ paragraph, 2 ^nd^ sentence: Is the word "no" really needed in this sentence?
6. In the “Discussion” section, 7 ^th^ paragraph, the last sentence: Is the word "first" really needed in this sentence?

Is the work clearly and accurately presented and does it cite the current literature?

Yes

If applicable, is the statistical analysis and its interpretation appropriate?

Not applicable

Are all the source data underlying the results available to ensure full reproducibility?

Yes

Is the study design appropriate and is the work technically sound?

Partly

Are the conclusions drawn adequately supported by the results?

Yes

Are sufficient details of methods and analysis provided to allow replication by others?

Partly

Reviewer Expertise:

Chronic disease management

I confirm that I have read this submission and believe that I have an appropriate level of expertise to confirm that it is of an acceptable scientific standard.

# Response

**Published:** 2022-03-19

**Authors:** Eva Cooney

**Affiliations:** National University of Ireland, Galway, Ireland

Dear Dr Puspitasari,

 Thank you very much for your review and detailed feedback on this article. I appreciate the time and consideration you have given. I have considered all the points you have made and will update the article accordingly. Please find specific answers to each point here:

 1. Recruitment via the IPU and PharmaBuddy happened at one time point at the beginning of the study but recruitment via social media and local pharmacies was more continuous. Data collection and analysis occurred alongside this and when no new themes were emerging, there was no further push for recruitment. I acknowledge this is not clear in the methods and will update to clarify. 

 2. It is challenging to identify the exact number of recruitment invitations as the IPU did not disclose the number of emails sent. As for the posts on social media, it is difficult to quantify how many people viewed these. However, I will update the article to explain this and include the numbers of letters delivered to pharmacies.

 3. The incorrect apostrophe is noted, thank you.

 4. 'They're' in this quote refers to the information days mentioned in the preceding sentence. 

 5. I believe the 'no' in this sentence is necessary as it captures the fact that some pharmacists were not aware of DSE at all. 

 6. I agree, the word 'first' is not needed here and I will remove, thank you. 

 Kind regards,

 Eva Cooney

# Peer review: Reviewer response for version 1

**DOI:** 10.21956/hrbopenres.14335.r29101

**Published:** 2021-03-23

**Authors:** Debbie Cooke

**Affiliations:** 1 School of Health Sciences, University of Surrey, Guildford, UK

This is a novel, qualitative interview study conducted with pharmacists based in Ireland to explore their views of their role in supporting diabetes education and self-management. It is very well written and the role of pharmacists in this area is under-researched. Hence, the study is valuable. I wonder if a little more can be drawn out about any differences between type 1 and 2 populations in the pharmacists' role in this area. What did the pharmacists think about this? Otherwise, I have no other suggestions or recommendations for improvement of the manuscript.

Is the work clearly and accurately presented and does it cite the current literature?

Yes

If applicable, is the statistical analysis and its interpretation appropriate?

Not applicable

Are all the source data underlying the results available to ensure full reproducibility?

Yes

Is the study design appropriate and is the work technically sound?

Yes

Are the conclusions drawn adequately supported by the results?

Yes

Are sufficient details of methods and analysis provided to allow replication by others?

Yes

Reviewer Expertise:

Health psychology and self-management of long-term conditions, particularly diabetes.

I confirm that I have read this submission and believe that I have an appropriate level of expertise to confirm that it is of an acceptable scientific standard.

# Response

**Published:** 2021-04-09

**Authors:** Eva Cooney

**Affiliations:** National University of Ireland, Galway, Ireland

Thank you Debbie for taking the time to review our paper and for your positive comments. Regarding your suggestion about drawing out any differences between type 1 and 2 populations, unfortunately this is not possible as such differences were not explored within the pharmacist interviews. I acknowledge this would have been a valuable addition to the paper, as pharmacists' experiences and interactions with people with diabetes likely do differ across these populations. Thank you for raising it, I will add this as a limitation of the study and highlight as an area for further exploration. 

 Kind regards,

 Eva Cooney
